# Epidemiology and Management of Cysticercosis and *Taenia solium* Taeniasis in Europe, Systematic Review 1990–2011

**DOI:** 10.1371/journal.pone.0069537

**Published:** 2013-07-29

**Authors:** Lorenzo Zammarchi, Marianne Strohmeyer, Filippo Bartalesi, Elisa Bruno, José Muñoz, Dora Buonfrate, Alessandra Nicoletti, Héctor Hugo García, Edoardo Pozio, Alessandro Bartoloni, The COHEMI Project Study Group

**Affiliations:** 1 Infectious Disease Unit, Department of Experimental and Clinical Medicine, University of Florence School of Medicine, Florence, Italy; 2 SOD Malattie Infettive e Tropicali, Azienda Ospedaliero-Universitaria Careggi, Florence, Italy; 3 Department G.F. Ingrassia, Section of Neurosciences, University of Catania, Catania, Italy; 4 Servicio de Medicina Tropical y Salud Internacional, Centre de Recerca en Salut Internacional de Barcelona, Hospital Clínic-Universitat de Barcelona, Barcelona, Spain; 5 Centre for Tropical Diseases, Sacro Cuore Hospital, Via Don Sempreboni, Negrar (Verona), Italy; 6 Cysticercosis Unit, Instituto de Ciencias Neurologicas, Department of Microbiology, Universidad Peruana Cayetano Heredia, Lima, Peru; 7 Department of Infectious, Parasitic and Immunomediated Diseases, Istituto Superiore di Sanità, Rome, Italy; Vanderbilt University, United States of America

## Abstract

**Background:**

Cysticercosis is caused by the invasion of human or pig tissues by the metacestode larval stage of *Taenia solium.* In Europe, the disease was endemic in the past but the autochthonous natural life cycle of the parasite is currently completed very rarely. Recently, imported cases have increased in parallel to the increased number of migrations and international travels. The lack of specific surveillance systems for cysticercosis leads to underestimation of the epidemiological and clinical impacts.

**Objectives:**

To review the available data on epidemiology and management of cysticercosis in Europe.

**Methods:**

A review of literature on human cysticercosis and *T. solium* taeniasis in Europe published between 1990–2011 was conducted.

**Results:**

Out of 846 cysticercosis cases described in the literature, 522 cases were autochthonous and 324 cases were imported. The majority (70.1%) of the autochthonous cases were diagnosed in Portugal from 1983 and 1994. Imported cases of which 242 (74.7%) diagnosed in migrants and 57 (17.6%) in European travellers, showed an increasing trend. Most of imported cases were acquired in Latin America (69.8% of migrants and 44.0% of travellers). The majority of imported cases were diagnosed in Spain (47.5%), France (16.7%) and Italy (8.3%). One third of neurosurgical procedures were performed because the suspected diagnosis was cerebral neoplasm. Sixty eight autochthonous and 5 imported *T. solium* taeniasis cases were reported.

**Conclusions:**

Cysticercosis remains a challenge for European care providers, since they are often poorly aware of this infection and have little familiarity in managing this disease. Cysticercosis should be included among mandatory reportable diseases, in order to improve the accuracy of epidemiological information. European health care providers might benefit from a transfer of knowledge from colleagues working in endemic areas and the development of shared diagnostic and therapeutic processes would have impact on the quality of the European health systems.

Key words: cysticercosis, neurocysticercosis, *Taenia solium*, taeniasis, Europe, travellers, migrants.

## Introduction

Cysticercosis is due to the invasion of human or pig tissues by the metacestode larval stage of *Taenia solium* [1], a helminth that may reach of up to 8 m in length in the adult stage. *T. solium* is a zoonotic cestode which has a complex two host life cycle [1]. Humans are the only definitive hosts, harbouring the adult tapeworm in the gut without having significant symptoms [2]. The adult tapeworm is acquired by eating raw or undercooked pork meat containing cysticerci [1]. Adult tapeworms have a life span of some years during which they produce millions of eggs which are intermittently released in the environment with the faeces [3]–[5]. Humans and pigs acquire cysticercosis ingesting *T. solium* eggs by the fecal-oral route [1]. After the ingestion, embryos contained in the eggs are released, cross the intestinal mucosa and are then transported by the circulatory system and dispersed throughout the body producing cysts mainly in the central nervous system (CNS) and in striated muscles [1]. Pigs are usually infected when reared in areas lacking adequate sanitary infrastructure where they can feed on human faeces [6].

Humans can acquire cysticercosis ingesting *T. solium* eggs released by themselves (autoinfestation) or by another tapeworm carrier living with them or involved in the preparation of food [7], [8]. In humans, the CNS is the most frequent localization of cysts which cause neurocysticercosis (NCC) [1]. NCC is considered to be the most common parasitic infection of the human nervous system and the most frequent preventable cause of epilepsy in the developing world [9] being responsible for about 30% of cases of epilepsy in low resources countries [10].

In several countries of Latin America, Africa and Asia, cysticercosis is highly endemic and linked to poverty, ignorance, lack of suitable diagnostic and management capacity and appropriate prevention and control strategies [9]. In these areas, cysticercosis leads to a very high economical burden related to losses in the meat industry for porcine cysticercosis, hospitalization costs for NCC and reduced productivity of people affected by NCC [2], [11], [12]. The situation is quite different in high resources countries such the United States and Europe. In the United States, cysticercosis has always been predominantly an imported disease and the number of observed cases increased from the 1970s onwards following the rise of immigration and the wide availability of the computerized tomography (CT) scan [13]. However in the period 1954–2005, 78 autochthonous cases have been reported in USA due to transmission from asymptomatic tapeworm carriers, who most likely acquired the infection abroad, to their household contacts [14].

In Europe, the disease was endemic in the past [15], but the natural life cycle of the parasite is currently completed very rarely thanks to the introduction of meat inspection, the progressive improvement of the pig husbandry, of the hygienic and socio-economic conditions and consumers education [15], [16]. Autochthonous cases of human and porcine cysticercosis are currently reported very rarely in Europe [17], [18], while human imported cases emerged in the last two decades only when immigration from low resource countries towards this continent became consistent [19]. In Europe, cysticercosis is not covered by specific surveillance systems. The epidemiology of the taeniasis/cysticercosis in high resources countries is probably changing, but the lack of reliable data is one of the major obstacles to understand the magnitude of the problem and the relevance of the disease is probably underestimated.

COHEMI (COordinating resources to assess and improve HEalth status of MIgrants from Latin America) is a three-year collaborative project supported by the European Commission under the Health Cooperation Work Programme of the 7th FRAMEWORK PROGRAMME (GA-261495) launched in 2011 (http://www.cohemi-project.eu/). The consortium brings together ten partners, six from Europe and four from Latin America (LA). One of the main objectives of the project is to provide a reliable estimate of the burden of selected Neglected Tropical Diseases in countries of origin and in different groups of migrants in Europe.

The aim of this study is to review, scientific and grey literature on the epidemiology and management of autochthonous and imported cases of cysticercosis and *T. solium* taeniasis in Europe.

## Methods

A review of indexed and grey literature on cysticercosis and *T. solium* taeniasis observed in Europe and published during the period 1990–2011 (July) was conducted. The data analysis focused on incidence and prevalence in the general population or special group of subjects, age and sex, infection rates, major clinical aspects, and country of infection.

The investigation covered 45 European countries, namely Albania, Andorra, Austria, Belarus, Belgium, Bosnia Herzegovina, Bulgaria, Croatia, Cyprus, Czech Republic, Denmark, Estonia, Finland, France, Germany, Greece, Hungary, Iceland, Ireland, Italy, Latvia, Lithuania, Luxemburg, Macedonia, Malta, Monaco, Moldova, Montenegro, Netherlands, Norway, Poland, Portugal, Romania, Russia, San Marino, Serbia, Slovakia, Slovenia, Spain, Sweden, Switzerland, Turkey, Ukraine, United Kingdom and Vatican State.

### Search

The following search strategy was adopted in PubMed (http://www.ncbi.nlm.nih.gov/pubmed/), Embase (http://embase.com/home), The Cochrane Library (http://www.thecochranelibrary.com/): (cysticerc* OR neurocysticerc* OR cisticerc* OR neurocisticerc* OR taenia* OR tenia* OR tapeworm) AND (Albania OR Andorra OR Austria OR Belarus OR Belgium OR Bosnia OR Herzegovina OR Bulgaria OR Cyprus OR Czech Republic OR Denmark OR Estonia OR Finland OR France OR Germany OR Greece OR Hungary OR Ireland OR Italy OR Latvia OR Lithuania OR Luxemburg OR Malta OR Monaco OR Moldova OR Netherlands OR Poland OR Portugal OR Romania OR San Marino OR Serbia OR Slovakia OR Slovenia OR Spain OR Sweden OR United Kingdom OR Ukraine OR Switzerland OR Norway OR Russia OR Croatia OR Montenegro OR Iceland OR Turkey OR Macedonia OR Vatican State OR Europe) AND (Humans[Mesh] AND (“1990/01/01”[PDAT] : “2011/07/31”[PDAT])). The search has been performed in August 2011. The online abstract book database of European Congress of Clinical Microbiology and Infectious Diseases (ECCMID, http://www.blackwellpublishing.com/eccmid/) from 2000 to 2010 were also screened. Given the characteristics of the online abstract book database of ECCMID, it was queried using the following keywords “cysticercosis”, “neurocysticercosis” and “*Taenia solium*”. Moreover, additional published and unpublished studies from the authors were included together with data from the Ministry of Health (MoH) of the European authors’ countries (Spain and Italy) concerning the number of hospitalizations for cysticercosis in the national territories according to ICD9CM codes, since very few documents reported epidemiological data concerning cysticercosis/*T. solium* taeniasis in Europe.

### Selection

The authors screened articles found by electronic search and evaluated their appropriateness based on title and abstract according to the established criteria. Exclusion criteria were: 1) studies concerning wrong agent (for example *Taenia saginata* or *Echinococcus* spp.); 2) reviews, letters or editorials without original data; 3) papers based on data only obtained through studies aiming to evaluate the performance of laboratory tests; 4) studies concerning animals only; 5) studies of European investigator outside the continent; 6) duplicated data; 7) articles with full texts written in languages other than that at least one of the members of the team could read and understand (namely English, French, Italian, German and Spanish). If the eligibility of the documents could not be ascertained according to the abstract and title only, the full text was analyzed to exclude or include the document. Moreover, in order to reduce possible underestimation of cysticercosis/*T. solium* taeniasis burden in some countries (mainly Eastern European countries), articles with an available English abstract, initially excluded due to the language of the full text, were selected as well.

### Extraction

Data from epidemiological studies were summarized, while data from case reports and case series were extracted using a standardized electronic form in which the main characteristics of study, clinical and epidemiological features of subjects with cysticercosis and/or *T. solium* taeniasis were recorded. The form included all the information needed to ascertain whether cases of cysticercosis and of *T. solium* satisfied the definition of definitive, probable or possible case (reported below). Concerning English abstracts of articles initially excluded due to language limits, only information contained in the abstract and referred to the number of cases reported and the most likely place of infection was extracted.

Cases of cysticercosis and *T. solium* taeniasis retrieved by the search were classified according to the following definitions:

- *Definitive or probable case of neurocysticercosis:* according to the criteria shown in Table 1, which were proposed by Del Brutto [20]. A case confirmed through Polymerase Chain Reaction (PCR) on bioptic specimen was also considered definitive;
- *Definitive case of cysticercosis outside of CNS:* case that was histologically confirmed by the presence of one or more cysticerci or PCR confirmed;
- *Possible case of cysticercosis or neurocysticercosis:* case reported by the authors as “cases of neurocysticercosis or cysticercosis”, but not fulfilling the criteria of definitive or probable case (for example if sufficient data to classify the case as definitive or probable were not available);
- *Confirmed case of* T. solium *taeniasis: T. solium* taeniasis case in which the diagnosis was based on the detection of gravid proglottids with information on the number of the uterine branches or case in which the diagnosis of *Taenia* spp. was done in a patient with cysticercosis or NCC;
- *Possible case of* T. solium *taeniasis: T. solium* taeniasis case reported by the authors as “cases of *T. solium* taeniasis”, but not fulfilling the criteria of confirmed case;
- *Autochthonous case*: case diagnosed in a subject without history of travel to or living in high endemic regions (Latin America, Asia or Africa). A case in which the subject travelled in Europe was considered autochthonous;
- *Imported case*: case diagnosed in a subject with a history of travel or long stay in a endemic region (Latin America, Asia or Africa).

**Table 1:** Diagnostic criteria and degree of certainty for the diagnosis of neurocysticercosis according to Del Brutto et al. [20].

Diagnostic criteria for neurocysticercosis	
Absolute	1. Histological demonstration of the parasite from biopsy of a brain or spinal cord lesion
	2. Cystic lesions showing the scolex on CT or MRI
	3. Direct visualization of subretinal parasites by funduscopic examination
Major	1. Lesions highly suggestive of neurocysticercosis on neuroimaging studies*
	2. Positive serum EITB† for the detection of anticysticercal antibodies
	3. Resolution of intracranial cystic lesions after therapy with albendazole or praziquantel
	4. Spontaneous resolution of small single enhancing lesions‡
Minor	1. Lesions compatible with neurocysticercosis on neuroimaging studies§
	2. Clinical manifestations suggestive of neurocysticercosis\
	3. Positive CSF ELISA for detection of anticysticercal antibodies or cysticercal antigens
	4. Cysticercosis outside the CNS¶
Epidemiological	1. Evidence of a household contact with *Taenia solium* infection
	2. Individuals coming from or living in an area where cysticercosis is endemic
	3. History of frequent travel to disease endemic areas
**Degree of certainty for the diagnosis of neurocysticercosis** **	
Definitive	1. Presence of one absolute criterion
	2. Presence of two major plus one minor and one epidemiologic criterion
Probable	1. Presence of one major plus two minor criteria
	2. Presence of one major plus one minor and one epidemiologic criterion
	3. Presence of three minor plus one epidemiologic criterion

**Footnotes:**

CT: Computed Tomography, MRI: Magnetic Resonance Imaging, EITB: Enzyme-linked immunoelectrotransfer blot assay, CSF: Cerebrospinal Fluid, ELISA: Enzyme-linked immunosorbent assay, CNS: Central Nervous System.

* CT or MRI showing cystic lesions without scolex, enhancing lesions, or typical parenchymal brain calcifications.

† Enzyme-linked immunoelectrotransfer blot assay using purified extracts of *Taenia solium* antigens, as developed by the Centers for Disease Control and Prevention (Atlanta, GA).

‡ Solitary ring-enhancing lesions measuring less than 20 mm in diameter in patients presenting with seizures, a normal neurologic examination, and no evidence of an active systemic disease.

§ CT or MRI showing hydrocephalus or abnormal enhancement of the leptomeninges, and myelograms showing multiple filling defects in the column of contrast medium.

\ Seizures, focal neurologic signs, intracranial hypertension, and dementia.

¶ Histologically confirmed subcutaneous or muscular cysticercosis, plain X-ray films showing “cigar-shaped” soft-tissue calcifications, or direct visualization of cysticerci in the anterior chamber of the eye.

** The presence of two different lesions highly suggestive of neurocysticercosis on neuroimaging studies should be considered as two major diagnostic criteria. However, positive results in two separate types of antibody detection tests should be interpreted only on the basis of the test falling in the highest category of diagnostic criteria.

## Results

**Figure 1:**
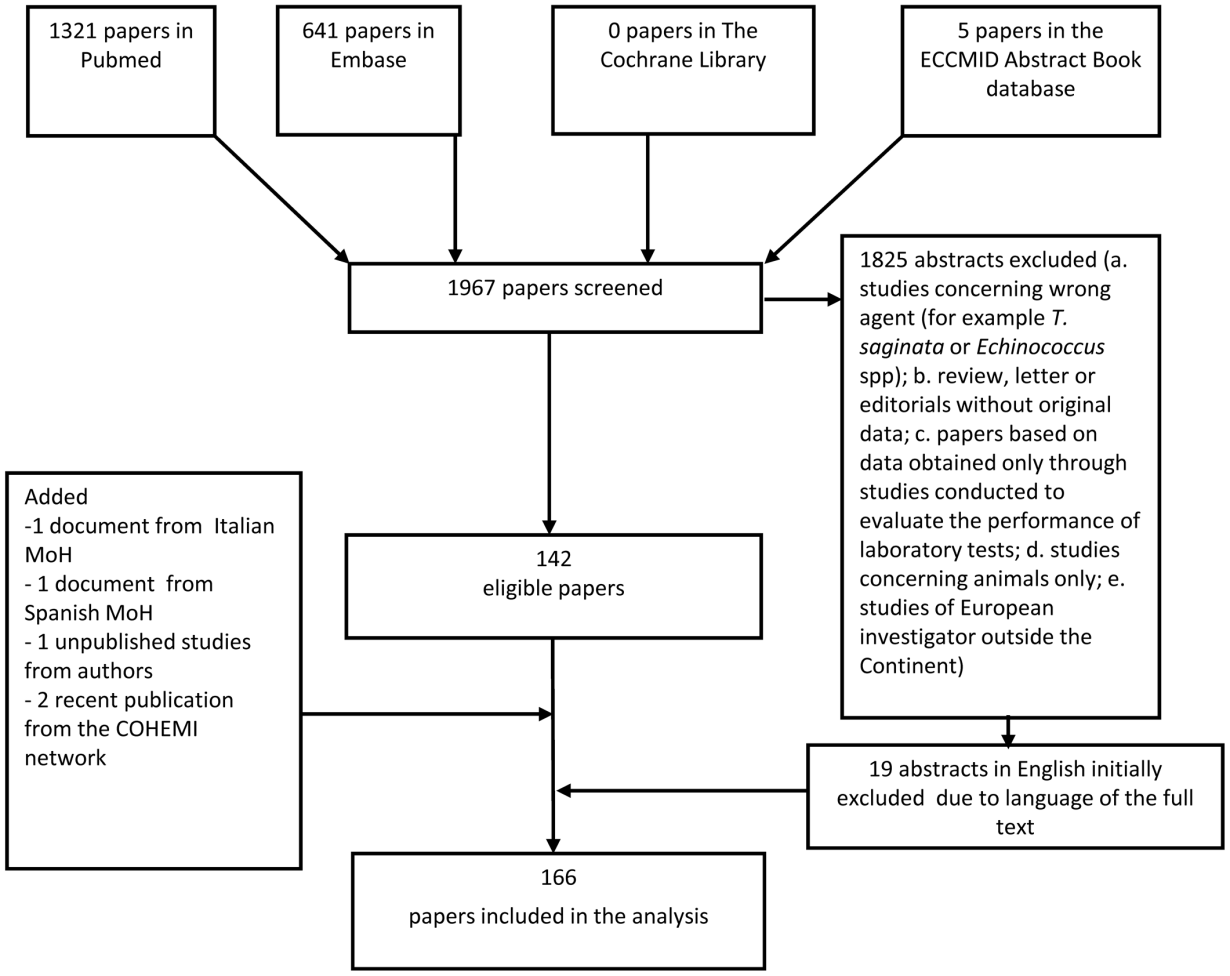
Flow diagram showing the number of papers identified in each database and the review process. **Legend:** ECCMID = European Congress of Clinical Microbiology and Infectious Diseases; MoH = Ministry of Health; COHEMI = COordinating resources to assess and improve HEalth status of MIgrants from Latin America.

The flow diagram in Figure 1 shows the number of papers identified in each database and the review process. Out of 1967 screened documents, 166 were included: 40 on autochthonous cysticercosis [17], [21]–[58], 7 on both autochthonous and imported cysticercosis [19], [59]–[64], 16 on autochthonous *T. solium* taeniasis [65]–[80], 97 on imported cysticercosis [81]–[176], [Bartoloni A. unpublished], 4 on imported cysticercosis and *T. solium* taeniasis [7], [177]–[179], and 2 on hospitalization for cysticercosis in Spain and Italy [180], [181].

### Autochthonous Human Cysticercosis in Europe

Information on autochthonous cysticercosis originated from 47 papers [17], [19], [21]–[64]. According to a three year prospective study on the etiology of adult-onset epilepsy, carried out in Extremadura (Spain), 6.3% (5 of 80) of new cases of epilepsy were related to neurocysticercosis [57]. According to a seroprevalence study on 770 patients with epilepsy resident in Croatia, the seroprevalence was 1.5% using an ELISA test confirmed by an EITB (Enzyme-linked Immunoelectrotransfer Blot) [182]. The other studies were case reports or case series (Tables 2, 3, 4 and Figure S1).

**Table 2:** Selected studies reporting autochthonous and imported cases of cysticercosis in Europe (1990–2011 July).

	Autochthonous	Imported
**Number of studies**	46	108
**Date of publication**		
1990–1994	14/46 (30.4%)	16/108 (14.8%)
1995–1999	6/46 (13.0%)	20/108 (18.5%)
2000–2004	14/46 (30.4%)	28/108 (25.9%)
2005–2009	11/46 (23,9%)	35/108 (32.4%)
2010–2011 July	1/46 (2.1%)	9^1^/108 (8.3%)
**First author field of activity**		
Neurology	15/44 (34.1%)	16/103 (15.5%)
Neurosurgery	8/44 (18.2%)	11/103 (10.7%)
Infectious Diseases	4/44 (9.1%)	19/103 (18.4%)
Pathology or Microbiology or Parasitology	4/44 (9.1%)	9/103 (8.7%)
Pediatrics	1/44 (2.3%)	11/103 (10.7%)
Neuroradiology	2/44 (4.5%)	7/103 (6.8%)
Ophtalmology	1/44 (2.3%)	7/103 (6.8%)
Other	9/44 (20.4%)	23/103 (22.3%)

**Footnotes:**

1. One study is still unpublished [Bartoloni A. unpublished].

**Table 3:** Main features of autochthonous and imported cases of cysticercosis in Europe (1990–2011 July).

	Autochthonous	Imported
**Number of cases**	522^1^	324^2^
**Case diagnostic certainty**		
definitive	33/496 (6.7%)	127/292 (43.5%)
probable	29/496 (5.8%)	66/292 (22.6%)
possible	434/496 (87.5%)^3^	99/292 (33.9%)
**Male sex**	85/189 (45%)	144/236 (61%)
**Mean age**	46.7±16.1	32.0±14.2
**Main symptoms**		
asymptomatic	182/384 (47.4%)	12/236 (5.1%)
epilepsy	166/384 (43.2%)	162/236 (68.6%)
severe headache	14/384 (3.6%)	39/236 (16.5%)
others	22/384 (5.7%)	23/236 (9.7%)
**Main localization of cysts**		
Central Nervous System (CNS)	492/496 (99.2%)	276/292 (94.5%)
eye	1/496 (0.2%)	10/292(3.4%)
skin or soft tissues	3/496 (0.8%)	6/292 (2%)
**Parasitological examination of stool showing taeniasis**	0/30 (0%)	5/133 (3.8%)
**Neuroimages pattern** 4		
cyst with scolex	3/276 (1.1%)	38/188 (20.2%)
cysts without scolex	75/276 (27.2%)	54/188 (28.7%)
enhancing lesions	23/276 (8.3%)	49/188 (26%)
calcifications	170/276 (61.6%)	65/188 (34.6%)
hydrocephalous	9/276 (3.3%)	14/188 (7.4%)
abnormal enhancement of leptomeninges	3/276 (1.1%)	-
others	2/276 (0.7%)	9/188 (4.8%)
**Serology**		
positive on serum^5^	60/94 (63.8%)	142/214 (66.4%)
positive on cerebrospinal fluid^6^	11/16 (68.7%)	48/69 (69.6%)
**Treatment**		
surgery only	23/115 (20%)	19/254 (7.5%)
surgery+antiparasitic	23/115 (20%)	32/254 (12.6%)
antiparasitic only	59/115 (51.3%)	172/254 (67.7%)
neither surgical nor antiparasitic treatment	10/115 (8.7%)	31/254 (12.2%)
**Indication for surgery (in neurocysticercosis)**		
ventriculo-peritoneal shunt for hydrocephalous	8/29 (27.6%)	16/46 (34.8%)
poor response to medical treatment	1/29 (3.4%)	2/46 (4.3%)
excisional or incisional biopsy for suspected CNS neoplasm	12/29 (41.4%)	15/46 (32.6%)
spinal cyst	3/29(10.4%)	3/46 (6.5%)
resections of ocular lesion	1/29 (3.4%)	6/46 (13%)
excision of intraventricular cyst	2/29 (6.9%)	3/46 (6.5%)
giant subarachnoid cyst	2/29 (6.9%)	1/46 (2.2%)

**Notes:**

1 Cases published between 1990 and 2011 July but referred to cases diagnosed between 1980 and 2006. The majority (366, 70.1%) were diagnosed in Portugal from 1983 to 1994 [34], [64].

2 Cases published between 1990 and 2011 July but referred to case diagnosed between 1981 and 2009.

3 Among this group, 348 subjects reported by Monteiro [34] were classified as “true” or probable, according to other criteria proposed by the same author in a previous paper [203].

4 Each subject may have more than one neuroimaging pattern.

5 EITB was used in 7 autochthonous and in 41 imported cases with positive serology on serum.

6 EITB was used in 5 autochthonous and in 9 imported cases with positive serology on CSF.

**Table 4:** Autochthonous and imported cases of cysticercosis in Europe by country of diagnosis (1990–2011 July).

	Autochthonous	Imported
**Number of cases**	522	324
**Country of diagnosis**		
Austria	1/522 (0.2%)	1/324 (0.3%)
Belgium	–	1/324 (0.3%)
Croatia	8/522 (1.5%)	–
Czech Republic	–	10/324 (3.1%)
Denmark	–	1/324 (0.3%)
Finland	–	1/324 (0.3%)
France	2/522 (0.4%)	54/324 (16.7%)
Germany	7/522 (1.3%)	15/324 (4.6%)
Greece	3/522 (0.6%)	–
Hungary	–	2/324 (0.6%)
Ireland	–	2/324 (0.6%)
Italy	7/522 (1.3%)	27/324 (8.3%)
Latvia	1/522 (0.2%)	1/324 (0.3%)
Luxemburg	1/522 (0.2%)	–
Norway	1/522 (0.2%)	3/324 (0.9%)
Poland	8/522 (15.3%)	–
Portugal	366/522 (70.1%)	20/324 (6.2%)
Spain	35/522 (6.7%)	154/324 (47.5%)
Serbia	78/522 (14.9%)	–
Switzerland	2/522 (0.4%)	4/324 (1.2%)
The Netherland	–	4/324 (1.2%)
Turkey	1/522 (0.2%)	–
United Kingdom	1/522 (0.2%)	24/324 (7.4%)

**Notes:**

Cases reported from Austria [39], Luxemburg [23], Norway [59], Switzerland [29], two case from Germany [31], [38] and one case from Spain [61] were diagnosed in subjects who travelled to or resided in other European countries. Concerning 348 cases reported from Portugal [34], the authors do not specify whether all cases are autochthonous, but state in the introduction that the catchment area of the hospital is highly endemic (district of Oporto and north inland).

No cases were reported from Albania, Andorra, Belarus, Bosnia and Herzegovina, Bulgaria, Cyprus, Estonia, Lithuania, Malta, Monaco, Moldova, Romania, San Marino, Slovakia, Slovenia, Sweden, Ukraine, Russia, Montenegro, Iceland, Macedonia, Vatican State.

### Autochthonous T. solium taeniasis in Europe

Only a confirmed case of *T. solium* taeniasis in a 25 years old Italian farmer, was documented [65]. In this case the authors explicitly specified that the diagnosis was based on observation of a several series of proglottids identified as *T. solium*. Forty-six cases of possible *T. solium* taeniasis were reported from Poland, with at least two cases per year in the period 1997–2008 [69]–[80], while no cases were reported in 2009 [68]. In Albania, from 1982 to 2002, 18 possible cases of *T. solium* were documented in 20 districts [67].The only available study on the prevalence of *T. solium* taeniasis was done in Szczecin Province (Poland) from 1994 to 1998. Only three *T. solium* taeniasis cases of 331 intestinal taeniasis, mainly due to *T. saginata* (n = 315), were documented. In this survey, prevalence of unspecified taeniasis decreased from 10.33/100,000 in 1994 to 3.52/100,000 in 1998 [66].

### Imported Human Cysticercosis in Europe

There are only two studies on anti-cysticerci IgG in adopted children [172], [179].

In Florence (Central Italy), 1090 adopted children, with a mean age of 5±3.4 years, were tested for anti-cysticerci IgG by a commercial EITB kit from 2001 to 2010, and the overall seroprevalence was 1% (0.3%, 1.8%, 1.6%, 0% in Asian, Latin American, African and European adopted children respectively). Mean age of seropositive children was higher than that of seronegative children (8±3.7 vs 5±3.3; p = 0.045). All seropositive infants (n = 11) underwent a neuroimaging study (CT scan or magnetic resonance imaging, MRI). Three presented abnormal findings compatible with neurocysticercosis, but only one had symptoms (epilepsy). The symptomatic child was a Peruvian female presenting also *T. solium* tapeworm in stool [179].

In Negrar (Northern Italy), serum samples from 842 adopted children were tested to detect anti-*T. solium* IgG by a commercial Enzyme-linked Immunosorbent Assay (ELISA) (91), or EITB (12) or both tests (739), from 2001 to 2008; of them, 87 sera tested positive at least by one test (76 sera, 9.0%, by ELISA; 12 sera, 1.6% by EITB). When both tests were used, positive results were discordant for all sera but one (1 ELISA+/EITB+; 64 ELISA+/EITB-; 11 ELISA+/EITB not done; 11 EITB+/ELISA-). All the 87 seropositive infants underwent a cerebral MRI. Eight (4 EITB+/ELISA-, 2 ELISA+/EITB not done, 2 ELISA+/EITB-) showed abnormal findings which were compatible with NCC, but only three had symptoms (epilepsy). The two children with ELISA+/EITB- sera showed a single enhancing lesion and a lesion compatible with a gliotic scar by MRI. The seroprevalence of anti-*T. solium* IgG in sera of Asian, Latin American, African and European adopted children was 10.0%, 7.6%, 5.2%, 10.7% by ELISA and 1.7%, 0.0%, 2.6%, 0.0% by EITB, respectively [172].

Out of 108 studies, 324 cases of imported cysticercosis were described [7], [19], [59]–[64], [81]–[170], [172], [174]–[179], [Bartoloni A., unpublished]; of them, 242 (74.7%) were migrants, 57 (17.6%) travellers, while no information was available for 25 cases (7.7%). Eleven (44.0%) travellers acquired the infection in Latin America, 10 (40.0%) in Asia (5 of whom in India), and 4 (16.0%) in Africa. The information was not available for 24 travellers or they had visited two or more endemic regions. Among migrants, 12 (5.1%) lived in more than one country before settling in Europe, therefore, it was not possible to know where they become infected. The majority of migrants (157; 69.8%) were Latin American (including 86 from Ecuador, 18 from Peru, and 17 from Bolivia), while the others were equally distributed between Africa (34, 15.1%, including 11 from Capo Verde and 6 from Madagascar), and Asia (34, 15.1%, including 18 from India). The time elapsed between the last exposure in an endemic country and the onset of symptoms was reported for 158 subjects. Among them, 33 (22.9%) developed symptoms within one year from the last exposure, 106 (73.6%) between 2 and 5 years, 11 (7.6%) between 6 and 10 years and 8 (5.6%) after 11 or more years (Table 2, 3, 4 and Figure S1). The majority of imported cases of cysticercosis were diagnosed in Spain (47.5%), France (16.7%), Italy (8.3%) and United Kingdom (7.4%). Seventy to 80% of imported cases in Spain and Italy were diagnosed after 2000 while 80 to 90% of imported cases in France and the United Kingdom were diagnosed before 2000.

### Imported T. solium taeniasis in Europe

Only five confirmed cases of *T. solium* taeniasis were documented in patients with cysticercosis. An African migrant died due to a disseminated cysticercosis following the ingestion of an adult *T. solium* tapeworm, a procedure suggested by an African healer to treat abdominal pain and nausea. Two mature segments of *T. solium* were recovered from faeces of this person [7]. The second case was a Latin American person with NCC in which the diagnosis was done through observation of gravid proglottids [177]. In the other three cases of *T. solium* taeniasis reported in the literature, the parasitological details of diagnosis were not specified but all were diagnosed in persons with NCC. Two cases were reported in migrants of unknown origin [178] and one in a Peruvian child [179].

**Hospitalization for Cysticercosis in Italy and Spain**

According to the Italian MoH, the number of hospitalizations for cysticercosis ranged between 40 and 53 per year in the period 2001–2010 [181]. The number of hospitalizations for migrants showed an increasing temporal trend while a decreasing trend was observed for Italians. From 2006 ongoing, the number of migrants hospitalized for cysticercosis has exceeded that of Italians. Using the number of hospitalizations by citizenship and by year as numerator and the number of residents in Italy by citizenship and by year as denominator [183], it was possible to estimate the hospitalization rate by 100,000 subjects in different groups. Considering the period 2001–2010, the higher mean hospitalization rate has been found in citizens from Latin America (5.9/100,000) and Asia (1.64/100,000), especially in Ecuadorian, Bolivian, Indian and Peruvian people (Table 5). According to the Spanish MoH from 2001 to 2009, the number of hospitalizations for suspected cases of cysticercosis ranged from 75 to 95 per year [180].

**Table 5:** Hospitalization for cysticercosis in Italy, 2001–2010 [181], [183].

	Number of hospitalizations for cysticercosis (2001–2010)	Estimated mean hospitalization rate for cysticercosis by citizenship (n/100,000) (2001–2010)
All residents in Italy	540	0.09
Italians citizens	276	0.05
Citizens of Latin American countries	132	5.90
Ecuadorians	51	13.70
Peruvians	35	5.57
Bolivians	17	42.79
Citizens of African countries	40	0.55
Citizens of Asian countries	78	1.64
Indians	64	10.18
Citizens of European countries (except Italy)	14	0.09

**References:**

162. Ministero della Salute Dipartimento della Qualità Direzione Generale Programmazione Sanitaria (2011) Dati estratti dalla banca dati delle schede di dimissione ospedaliera anni 2001–2010 per il progetto COHEMI. DGPROG 1.9.b/3. Italy.

163. Istituto Nazionale di Statistica (2011) Cittadini stranieri. Bilancio Demografico e popolazione residente straniera al 31 dicembre per sesso e cittadinanza.

## Discussion

The results of our findings, about epidemiology of *T. solium* taeniasis and cysticercosis in Europe, update those previously published by Overbosch et al. [184] and supplement those recently published by Del Brutto focusing on the epidemiology of NCC in Western Europe [185]. With respect to the latter review, our systematic research strategy included also grey literature and data from Eastern European countries, it was not limited to the CNS involvement, and focussed also on *T. solium* taeniasis. Moreover, data on the diagnostic and therapeutic management of the reported cases were also recorded.

Autochthonous cases of cysticercosis most of which were diagnosed in persons from the Mediterranean region (Iberian Peninsula, Italy and Balkans), are decreasing. In fact, most of these cases (366, 70.1%) were reported in Portugal from 1983 to 1994 [34], [64]. Persons who acquired cysticercosis in Europe were older than those who acquired cysticercosis abroad, and were more frequently asymptomatic, often presenting brain calcifications (the end-stage inactive lesion of cysticercosis) and without taeniasis. These clinical data suggest that these people acquired the infection in a remote past.

Our literature investigation does not allow to know whether the *T. solium* cycle is still active in Europe. Since *T. solium* taeniasis is acquired by eating insufficiently cooked pig meat with cysticerci, the presence in the European regions of porcine cysticercosis is the “condition sine qua non” to acquire *T. solium* taeniasis in Europe. The importation of cysticercus-infected pork can be excluded due to the veterinary controls, through meat inspection performed at slaughterhouse, and the circulation of cysticercus-infected pigs exists only in small and poor areas of the world where pigs are used only for local consumption and which do not trade at the international level.

Our search retrieved 68 cases of *T. solium* taeniasis in native European persons, the large majority of which (67.6%) have been reported from Poland. At the diagnosis, *T. solium* taeniasis may be confused with the very common *T. saginata* which is circulating with a 0.01–2% prevalence in Western Europe and with a higher prevalence in Eastern Europe [186]. By morphology, *T. solium* and *T. saginata* eggs are indistinguishable between them and the two Taeniidae species can be distinguished only by the count of the uterine branches in the proglottids or by the scolex which, however, is very rarely detectable [187]. Specific faecal antigen detection or PCR method are not yet performed in clinical practice and, to our knowledge, were never used for an epidemiological study in Europe.

To date in Western Europe, intensive indoor pig productions largely predominate. However, the increasing trend to produce organic pork from free-ranging pigs could increase the risk of *T. solium* transmission [188], and could represent a reason of concern for the control of cysticercosis in Europe. In 2007, a survey of the European Food Safety Authority, among European Union countries members, showed that porcine cysticercosis was still diagnosed in five countries (Austria, Estonia, Lithuania, Poland and Romania), no cases of porcine cysticercosis were reported in 9 countries (Belgium, Czech Republic, Denmark, Germany, Italy, Luxembourg, the Netherlands, Portugal and the United Kingdom), whereas no information was available for the other 11 EU countries [18]. This survey suggests that *T. solium* taeniasis is still circulating in Europe; however, these data should be carefully interpreted because porcine cysticercosis due to the larval stage of *Taenia hydatigena* can be misdiagnosed with the infection caused by larvae of *T. solium.* The absence of porcine cysticercosis recently reported in Portugal [18], where an hotspot of cysticercosis transmission was observed up to early nineties [34], suggests the possible interruption of transmission due to the modification of breeding conditions adopted in the recent years. Autochthonous cases of cysticercosis in Europe could also originate from *T. solium* carriers (migrant or traveller) who acquired taeniasis outside Europe as observed in the United States [8], [14]. In our search, we found only 5 cases of *T. solium* immigrant carriers. Consequently, we can speculate that the risk of spreading cysticercosis in Europe from an asymptomatic tapeworm carriers coming from abroad is quite low even if it cannot be excluded.

The larval stage of *Taenia crassiceps*, *Taenia multiceps* and *Taenia serialis* infecting canids as final host and rodents, rabbits, sheep and goats as intermediate host can sporadically affect humans in Europe. In some cases, neither pathological examination nor serology can differentiate these parasites from *T. solium* cysticerci [50]. Therefore, we cannot exclude that a portion of cases diagnosed as cysticercosis in Europe has been caused by other species of cestodes.

In Europe, the increasing number of migrants from low resource countries (74.7% of cysticercosis imported cases) and international travellers (17.6% of imported cases) resulted in an increase of cysticercosis. Most of imported cases originated from Latin America, where 44.0% of affected travellers and 69.8% of affected migrants acquired the infection. Most of individuals with imported disease developed symptoms after 2 to 5 years after migration. This finding is quite similar to that obtained in the classic epidemiological studies carried out in the sixties in English soldiers returning from India [189], confirming that the incubation period of cysticercosis, even if largely variable, is usually between 2 to 5 years.

The European countries where cysticercosis cases are more often imported are those with larger immigration such as Spain, France, Italy and the United Kingdom. Spain is the country reporting the highest number of imported cases of cysticercosis, probably because it hosts the largest number of Latin American migrants in Europe.

Considering both autochthonous and imported cases, neurologists, infectious disease specialists, neurosurgeons, laboratory specialists (microbiologists, parasitologists, pathologists), paediatrics and radiologists were the most often involved persons in the management of people with cysticercosis in Europe. This wide range of specialists confirms the need of knowledge in different settings to manage cysticercosis. NCC is certainly the parasite localization which is managed with more difficulties since there is not a gold standard test for the diagnosis but only diagnostic criteria and no treatment of choice but only recommendation to tailor the therapy case by case [190].

From the clinical point of view, NCC does not shows any pathognomonic signs or symptoms. On the contrary, it may present with nearly all neurological signs or symptoms [191], [192]. Neuroradiological images are pathognomonic only in few cases when the scolex is visible in the cyst (named “cyst with dot” image) [20]. Serological tests didn’t help too much. Several serological assays to detect specific antibodies have been used for decades with different and somewhat conflicting results [193], [194]. Currently, most centres use an EITB with purified glycoprotein antigens [195], which can be performed on serum samples or on cerebrospinal fluid (CSF) or use an ELISAs. The EITB sensitivity in serum samples is equal to or better than that in CSF samples [196]. Although EITB has 100% specificity and a sensitivity of 98% in patients with two or more cerebral lesions, up to 50% of patients with a single brain lesion or with only calcified parasites may test negative [197]–[199]. The main problem related to ELISA on serum is the poor specificity which is reported around 70% or less [194]. In any case, serological results must be carefully interpreted together with the other diagnostic criteria. So far, EITB positivity on serum is considered to be a major diagnostic criterion and ELISA positivity on CSF a minor diagnostic criterion [20]. Recently, a serum antigen detecting ELISA has been reported to have an acceptable sensitivity (83–100%) and specificity (84–96%) and proposed as an adjunctive tool for the diagnosing NCC [200].

The diagnostic delay can be huge. In one person, NCC was misdiagnosed with migraine, cluster headache, and viral meningitis for 29 years after the onset of symptoms [111]. Parenchymal NCC can be misdiagnosed with brain tumour and lead to avoidable surgery as in the two cases reported by Bouillot [163]. About one third of neurosurgical operations reported in the examined literature, were probably avoidable since the indication to surgery was not among that are commonly accepted [201]. In autochthonous and imported cases of cysticercosis, 41.4% and 32.6% of operations, respectively, were performed for suspected CNS neoplasm. This high rate of neurosurgical interventions can be attributed to the low awareness and experience in management of the disease and the inadequacy of preoperative diagnostic tools [43].

According to Ruiz [154], 22.8% (8 of 35) of persons received albendazole in spite of not having antiparasitic treatment indication. Finally, it should be stressed that the treatment of NCC cannot be limited to surgery and/or antiparasitic drugs. The symptomatic therapy including anti-inflammatory drugs (corticosteroids), analgesics, adequate antiepileptic treatment as for other secondary epilepsies, represents the main measure in the management of NCC, however, these important treatment issues are rarely discussed in case reports [202].

Conclusions: in Europe we are currently observing the overlapping of two epidemiological pictures of cysticercosis/*T. solium* taeniasis: the autochthonous and the imported infections. Autochthonous cases are disappearing, while imported cases are rising as a consequence of travels and migrations. Given the lack of systematic epidemiological data collection and disease reporting, the magnitude of the phenomenon cannot be delineated. Moreover, it is unclear if cases of imported *T. solium* taeniasis are now generating autochthonous cases of cysticercosis but, according to our search, it is probably a limited problem. In order to get more information, policy makers should consider to include cysticercosis among mandatory reportable diseases. From the clinical point of view, NCC remains a challenge for clinicians despite their full access to all the available tools for diagnosis and management of the disease. The currently available diagnostic tests and treatment options are frequently insufficient and research activities in this field are strongly needed. Within a perspective of a south-north and north-south cooperation, European health care providers might benefit from a transfer of knowledge from expert colleagues working in endemic areas and the development of shared diagnostic process and therapeutic decisions would have impact on the European health systems quality.

## Supporting Information

Figure S1 Maps showing the geographical distribution by country of diagnosis of autochthonous and imported cases of cysticercosis reported in Europe (1990–2011 July). (TIF)

Table S1 PRISMA Checklist. (DOC)

Table S2 PRISMA Flowchart. (DOC)
