# Correction: Epidemiology and Management of Cysticercosis and *Taenia solium* Taeniasis in Europe, Systematic Review 1990–2011

**DOI:** 10.1371/annotation/1bcc3e5b-1159-412b-be86-b18d94515cc2

**Published:** 2013-12-19

**Authors:** Lorenzo Zammarchi, Marianne Strohmeyer, Filippo Bartalesi, Elisa Bruno, José Muñoz, Dora Buonfrate, Alessandra Nicoletti, Héctor Hugo García, Edoardo Pozio, Alessandro Bartoloni, The COHEMI Project Study Group

Figure S1 is a duplication of Figure 1. Please refer to the correct Figure S1 here: http://plosone.org/corrections/pone.0069537.s001.cn.tif

