# Supplementary figures and images for: Epidemiology and Management of Cysticercosis and Taenia solium Taeniasis in Europe, Systematic Review 1990–2011
Source: PLoS One. 2013 Jul 29;8(7):e69537. doi: 10.1371/journal.pone.0069537 (PMC3726635; doi:10.1371/journal.pone.0069537)

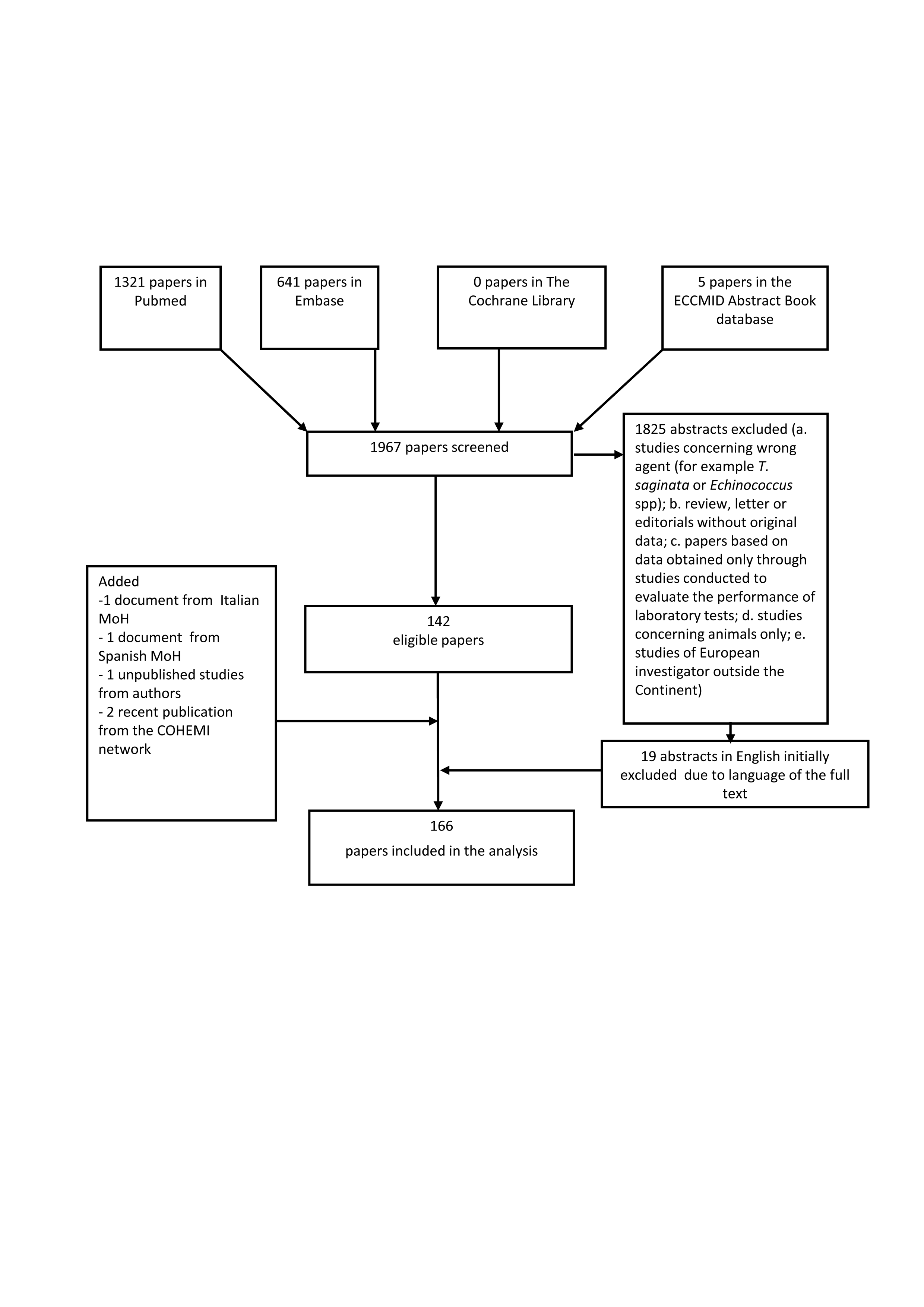

Supplement: Figure S1 — (TIF) [file pone.0069537.s001.tif]
