# Supplementary material for: Epidemiology and Management of Cysticercosis and Taenia solium Taeniasis in Europe, Systematic Review 1990–2011
Source: PLoS One. 2013 Jul 29;8(7):e69537. doi: 10.1371/journal.pone.0069537 (PMC3726635; doi:10.1371/journal.pone.0069537)
Supplement: Table S2 — (DOC) [file pone.0069537.s003.doc]

**Epidemiology and management of cysticercosis and *Taenia solium* taeniasis in Europe, systematic review 1990-2011**


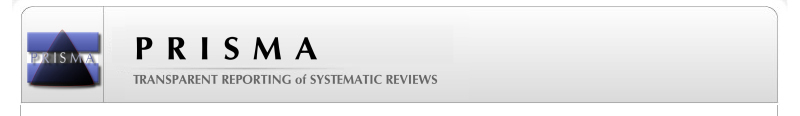
**Table S2: PRISMA 2009 Flow Diagram**

**Screening**

**Included**

**Eligibility**

**Identification**

Records identified through database searching
(n = 1967)

Additional records identified through other sources
(n = 5)

Records screened
(n = 1972)

Records excluded
(n = 1806)

Eligible documents (142 full texts, 19 abstracts and

5 additional records)

Studies included in qualitative and quantitative synthesis
(n = 166)
